# Rats that are predisposed to excessive obesity show reduced (leptin‐induced) thermoregulation even in the preobese state

**DOI:** 10.14814/phy2.14102

**Published:** 2019-07-24

**Authors:** Kathy C.G. de Git, Johannes A. den Outer, Inge G. Wolterink‐Donselaar, Mieneke C. M. Luijendijk, Erik Schéle, Suzanne L. Dickson, Roger A. H. Adan

**Affiliations:** ^1^ Brain Center Rudolf Magnus Department of Translational Neuroscience University Medical Center Utrecht Utrecht University Utrecht The Netherlands; ^2^ Institute for Neuroscience and Physiology The Sahlgrenska Academy at the University of Gothenburg Gothenburg Sweden

**Keywords:** Brown adipose tissue, leptin, leptin sensitivity, tail temperature, thermogenesis

## Abstract

Both feeding behavior and thermogenesis are regulated by leptin. The sensitivity to leptin's anorexigenic effects on chow diet was previously shown to predict the development of diet‐induced obesity. In this study, we determined whether the sensitivity to leptin's anorexigenic effects correlates with leptin's thermogenic response, and if this response is exerted at the level of the dorsomedial hypothalamus (DMH), a brain area that plays an important role in thermoregulation. Based on the feeding response to injected leptin on a chow diet, rats were divided into leptin‐sensitive (LS) and leptin‐resistant (LR) groups. The effects of leptin on core body, brown adipose tissue (BAT) and tail temperature were compared after intravenous versus intra‐DMH leptin administration. After intravenous leptin injection, LS rats increased their BAT thermogenesis and reduced heat loss via the tail, resulting in a modest increase in core body temperature. The induction of these thermoregulatory mechanisms with intra‐DMH leptin was smaller, but in the same direction as with intravenous leptin administration. In contrast, LR rats did not show any thermogenic response to either intravenous or intra‐DMH leptin. These differences in the thermogenic response to leptin were associated with a 1°C lower BAT temperature and reduced UCP1 expression in LR rats under ad libitum feeding. The preexisting sensitivity to the anorexigenic effects of leptin, a predictor for obesity, correlates with the sensitivity to the thermoregulatory effects of leptin, which appears to be exerted, at least in part, at the level of the DMH.

## Introduction

Obesity rates continue to rise in adults and children (Rezai‐Zadeh and Munzberg 2013), and there is a high and unexplained variability in the susceptibility for the development of obesity (Ruffin et al. 2004). Like humans, several rat strains show individual differences in the susceptibility for the development of diet‐induced obesity (DIO) (Levin and Dunn‐Meynell 2002; Levin et al. 2004; Ruffin et al. 2004; Tulipano et al. 2004; de Git et al. 2018), which provides opportunities to study preexisting vulnerability factors for DIO.

We (de Git et al. 2018) and others (Levin and Dunn‐Meynell 2002; Ruffin et al. 2004) previously showed that reduced sensitivity to leptin's anorexigenic effects is a preexisting vulnerability factor for DIO. Based on the feeding response to exogenously injected leptin on a chow diet, we divided Wistar rats into leptin‐sensitive (LS) and leptin‐resistant (LR) groups (de Git et al. 2018). LR rats were more prone to develop obesity on a free choice high‐fat high‐sucrose (fcHFHS) diet compared with LS rats, without eating more calories. In comparison to LS rats, LR rats showed a preexisting reduction in the activation of leptin‐induced signal transducer and activator of transcript 3 (pSTAT3), a marker for cellular leptin sensitivity (Bates et al. 2003; Gao et al. 2004), in the dorsomedial hypothalamus (DMH) but not the arcuate nucleus (ARC). While similar pSTAT3 activation in the ARC in LS and LR rats may explain why LR rats did not eat more, it is still unclear how the preexisting reduction in pSTAT3 activation in the DMH predisposes LR rats to exacerbated DIO.

Although there is evidence that leptin action in the DMH regulates energy balance by reducing food intake (Dodd et al. 2014; Enriori et al. 2011 but see Rezai‐Zadeh et al. 2014), leptin is particularly known to activate brown adipose tissue (BAT) thermogenesis via neurons in the DMH (Enriori et al. 2011; Rezai‐Zadeh and Munzberg 2013; Dodd et al. 2014; Jo and Buettner 2014; Rezai‐Zadeh et al. 2014). Several lines of evidence demonstrate a critical role for leptin signaling in the DMH in mediating BAT‐dependent thermogenesis: (1) Injection of leptin directly into the DMH increased BAT temperature (Enriori et al. 2011); (2) Leptin‐induced increases in BAT temperature were blocked by preinjection of a leptin receptor antagonist directly into the DMH (Enriori et al. 2011); (3) Selective activation of the leptin receptor (LepRb) expressing neurons within the DMH increased BAT and core body temperature (Rezai‐Zadeh et al. 2014); (4) Knock‐out of LepRb in a specific population of DMH neurons, expressing prolactin‐releasing peptide, blocked leptin‐induced increases in UCP1 and core body temperature (Dodd et al. 2014). However, leptin regulation of core body temperature appears not to arise exclusively from BAT thermogenesis, as BAT temperature did not always precede and exceed the increase in core body temperature evoked by leptin receptor signaling (Rezai‐Zadeh et al. 2014). More recently, it has been reported that, at least in ob/ob mice, systemic leptin injection leads to a pyrexic increase in core body temperature by reducing heat loss via the tail (Fischer et al. 2016b).

In this study, we aimed to unravel whether rats that are less sensitive to the anorexigenic effects of peripherally injected leptin, also show a reduced thermogenic response to peripheral leptin. Furthermore, to explore whether a reduced thermogenic response to peripheral leptin could be due to reduced cellular leptin signaling in the DMH (as opposed to, e.g., impaired leptin transport across the blood–brain barrier), we compared leptin regulation of thermogenesis after intravenous and also after intra‐DMH leptin injection between LS and LR rats fed regular chow. We also explored the contribution of BAT thermogenesis and heat loss via the tail to leptin's effect on core body temperature.

## Methods

### Animals

Adult male Wistar rats (Charles River, Sulzfeld, Germany) were individually housed in Plexiglas cages in a temperature controlled (21–23°C) and light controlled (lights on between 08.00 and 20.00 h) room. Rats had ad libitum access to pelleted rat chow (3.31 kcal/g; Special Diet Service, UK) and tap water, unless otherwise stated. All experiments were performed in accordance with Dutch laws (Wet op de Dierproeven, 1996) and European regulations (Guideline 86/609/EEC) and were approved by the Animal Ethics Committee of Utrecht University.

### Surgery

When the rats had reached a body weight of > 300 g, they underwent surgery to implant: (1) Intraarterial silicone catheters through the right jugular vein, according to the method of Steffens (Steffens 1969); (2) Stainless steel guide cannulas (26 GA, 9 mm; Plastics One, Roanoke, USA) bilaterally above the DMH (1 mm above the DMH, from bregma: anterior‐posterior: −2.50, medio‐lateral: ±2.10, dorso‐ventral: −8.60, at an angle of 10°, Paxinos and Watson, 1998, fourth edition). Cannulas were fixed to the skull with stainless steel screws and dental cement; (3) An intraabdominal dual transmitter (TL11M3F40‐TT, Data Science International (DSI), USA) with temperature‐sensing leads to the portal vein in the liver and interscapular BAT.

### Selection of leptin‐sensitive versus leptin‐resistant rats

To divide rats into two types of leptin responders, leptin sensitivity of each individual rat was determined twice, and the average response was taken. Rats were divided into two subgroups based upon their average feeding response at 1 h after leptin injection, as the variability was largest at this time point. Rats showing a reduction in food intake (percentage suppression < 100) were designated as LS, whereas rats showing no reduction or even an increase in food intake were designated as LR (percentage suppression ≥ 100) (de Git et al. 2018).

### Telemetric measurements

The home cage was placed on a receiver plate (DSI, USA) that received radiofrequency signals from the abdominal transmitter. The plate was connected to software (DSI) that recorded core body temperature, BAT temperature, and locomotor activity every 2 min. In seven rats, the battery of the transmitters was empty before the end of the experiment. These rats were therefore excluded from the telemetric measurements from the time point of the empty battery onwards.

To test the effect of leptin on body temperature, rats were food restricted for two consecutive days (10 gr chow per day at 16.00 h). The next morning at 9.00 h, leptin or vehicle was injected according to a Latin square design. Two hours later food was given back, and 24 h food intake was measured. The interval between the two test days of a Latin square design was 7–10 days. The effect of intravenous and intra‐DMH leptin (recombinant murine leptin, NHPP, USA) on body temperature was tested with two independent Latin square designs. For intravenous injections, leptin (250 μg/250 μL) or vehicle (phosphate‐buffered saline, PBS) was injected via the jugular vein cannula. Intra‐DMH leptin injections were performed through an injector (10 mm, 33GA, Plastic One) inserted into the guide cannula. Bilateral infusions (300 ηg leptin/300 ηL PBS over one minute) were performed using a syringe pump with the injectors left in place for another minute to prevent backflow. The effect of intra‐DMH leptin on food intake was tested during the same experiment as for the effect of intra‐DMH leptin on body temperature. Rats that were excluded from the temperature results due to telemetry issues were included in food intake measurements.

### Thermosensitive camera

**Figure 1:**
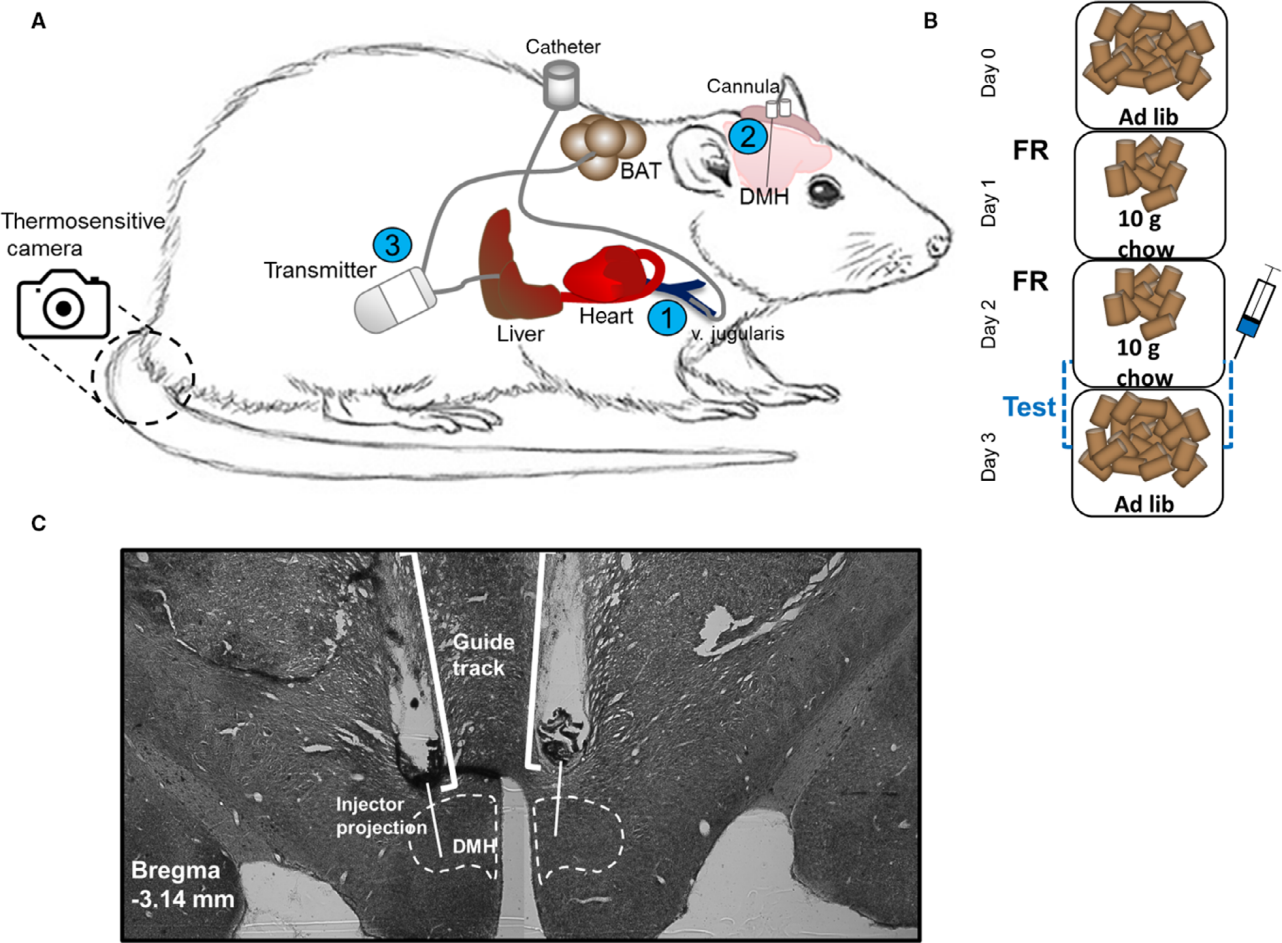
Animal model and experimental design. (A) Rats underwent surgery to implant: 1) A catheter in the jugular vein; 2) Local cannulas bilaterally above the DMH; 3) An intraabdominal telemetric transmitter with probes in the liver and brown adipose tissue (BAT). An infrared/thermosensitive camera was used to measure the tail base temperature. The region of interest for the tail base is indicated. (B) The thermogenic response to leptin was tested both via systemic injections through the jugular catheter (250 ug, i.v), and local infusions in the DMH (bilateral, 300 ng/300 nL/60sec). Rats were food restricted prior to injections to lower their body temperature. (C) Example of the anatomical verification of correctly placed DMH cannulas.

During test days, the impact of leptin on tail temperature was examined using a FLIR infrared/thermal camera (E60bx: Compact‐Infrared‐Thermal‐Imaging‐Camera; FLIR; West Malling, Kent, UK) (Fig. 1A).

### Postmortem analysis

The analysis of cannula placement revealed uni‐ or bilateral DMH cannula(s) misplacement in five rats (three LS rats and two LR rats). These rats were excluded from the intra‐DMH analyses.

The placement of the transmitter leads to the liver and interscapular BAT was also checked after sacrifice. All liver probes were placed correctly, but the BAT probe was misplaced in seven rats (three LS rats and four LR rats). These rats were therefore excluded from the BAT temperature analysis. BAT tissue was dissected and stored for subsequent UCP1 analysis (see supplementary text).

We aimed to perform all experiments in the same group of animals. Due to the technical limitations indicated above, it was not possible to include all animals in each temperature measurement. Table S1 gives an overview of the animals that were included in each temperature measurement and shows the reason for exclusion of the other rats.

### Statistical analysis

For differences in body temperature, activity, body weight, and caloric intake, two‐way repeated measures ANOVAs were performed with time as within‐subject variable and responder (LS/LR) as between‐subject variable. BAT UCP1 expression levels were compared between LS and LR rats with an independent t‐test. For fat mass analysis, a one‐way ANOVA was performed with responder (LS/LR) as a between‐subject variable. Feeding responses to leptin were assessed using a three‐way repeated measures ANOVA with time and treatment as within‐subject variables and responder (LS/LR) as between‐subject variable. Thermogenic responses to leptin were assessed using a two‐way ANOVA on the average data of temperature in the absence and the presence of food, respectively, with treatment as within‐subject variable and responder as between‐subject variable.

Mauchly's test of sphericity was used to test whether variances of the differences between treatment levels were equal. If the assumption of sphericity was violated, degrees of freedom were corrected using Greenhouse‐Geisser (GG) estimates of sphericity or Huynh–Feldt estimates of sphericity when the GG estimate was > 0,75. When appropriate, post hoc analyses were conducted using Student's t‐tests or pairwise Bonferroni comparisons. Each parameter was tested for normality with the Kolmogorov–Smirnov test. When data were not normally distributed, data were log transformed prior to statistical analyses.

Statistical analyses were conducted using SPSS 20.3 for Windows. The threshold for statistical significance was P < 0.05. The effects of leptin on thermoregulation were tested with one‐sided post hoc t‐tests, as leptin is generally accepted to increase body temperature (Enriori et al. 2011; Dodd et al. 2014; Rezai‐Zadeh et al. 2014) and was shown to reduce tail temperature (Fischer et al. 2016b). The main effects of responder on daily BAT and core body temperature, and BAT UCP1 levels were also tested one‐sided, as we expected lower temperatures and UCP1 levels in LR rats. Data are presented as mean ± SEM.

Additional details regarding methods can be found in supplementary information.

## Results

### Distinguishing two types of leptin responders on a chow diet

**Figure 2:**
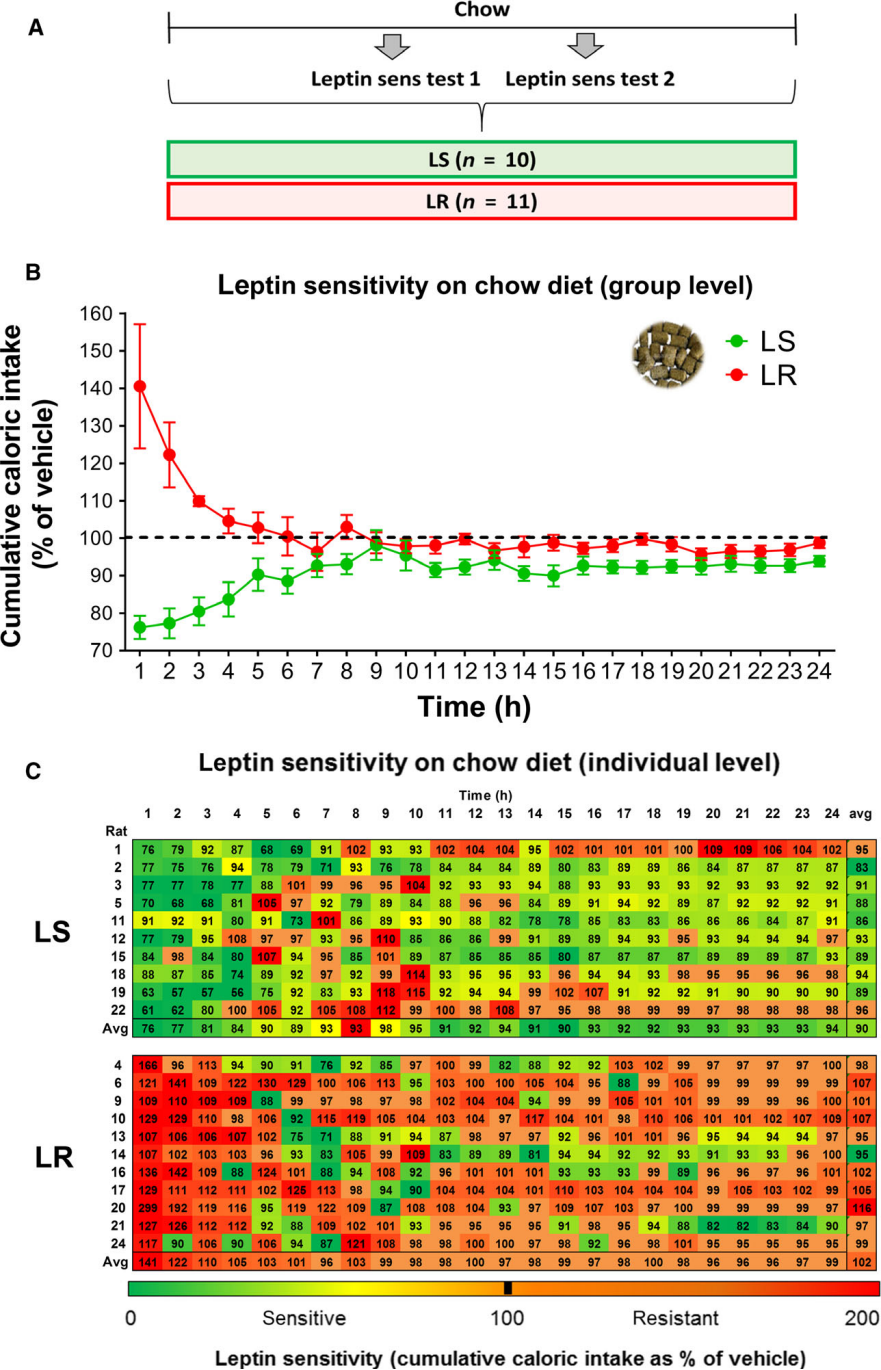
Individual 1–24 h leptin sensitivity in LS and LR rats fed a chow diet. Rats were divided into those showing a reduction in food intake during the first hour after intravenous leptin injection (leptin sensitive, LS) and those that did not change or increased their food intake with intravenous leptin at 1 h food intake (leptin resistant, LR). (A) Experimental design. Leptin sensitivity was measured by cumulative food intake after leptin injection normalized to vehicle food intake. Average leptin sensitivity of two tests is shown. Leptin sensitivity (B) at group level and (C) individual level; a heat plot of the relative level of sensitivity is shown at 1–24 h food intake for each individual rat (i.e., each row). The heat plot indicates the relative degree of leptin sensitivity at a particular time point in comparison with the other time points in the row. Data are shown as mean ± SEM; n = 10–11 per group

Rats were divided into leptin‐sensitive (LS, n = 10) and leptin‐resistant (LR, n = 11) groups based on their feeding response to leptin injection, normalized to baseline vehicle, at the first hour following injection (Fig. 2B and C), as described previously (de Git et al. 2018). The selection of LS and LR rats was based on the average leptin sensitivity of two independent leptin sensitivity tests (Fig. 2A). The food intake response to leptin at 1 h after injection ranged from −39.4% to −9.0% (−23.8% ± 3.1) in LS rats and from +0.7% to +98.6% (+40.6%±16.6) in LR rats. Further inspection of the leptin sensitivity patterns of LS and LR rats at 2–24 h following leptin injection revealed that LS and LR rats also differ in their leptin sensitivity at later time points, especially at 2–5 h following injection (Fig. 2B and C). LS rats significantly reduced their food intake following leptin injection at almost all time points. In contrast, LR rats did not show a leptin‐induced reduction in food intake over 24 h following injection (Fig. 2B and C). There was no difference in the amount of food eaten between LS and LR rats following vehicle injections (1 h: 12.8 ± 0.7 vs. 10.6 ± 1.2 kcal), and classification based on 1 h cumulative food intake (vehicle FI – leptin FI) resulted in the same distribution of LS and LR rats (data not shown).

In accordance with our previous findings (de Git et al. 2018), the distinct leptin sensitivity patterns in LS versus LR rats were not associated with differences in body weight, caloric intake, and adiposity on a chow diet (Fig. S1). The increased susceptibility for the development of obesity in LR rats was previously specifically shown after exposure to an obesogenic fcHFHS diet (de Git et al. 2018).

There is evidence that leptin signaling in the DMH mediates food intake (Enriori et al. 2011; Dodd et al. 2014). We therefore compared the feeding response to leptin after intravenous versus intra‐DMH injection (Fig. S2A–C). The DMH was not critically involved in leptin regulation of food intake suppression. This finding is in accordance with the contradictory effects of leptin signaling in the DMH on food intake in previous studies (Dodd et al. 2014; Enriori et al. 2011 vs. Rezai‐Zadeh et al. 2014). Since leptin action in the DMH has been shown to regulate energy balance particularly by increasing energy expenditure (Enriori et al. 2011; Dodd et al. 2014; Rezai‐Zadeh et al. 2014), we next focused on the comparison of (leptin regulation of) thermoregulation between LS and LR rats.

### LR rats show lower UCP1 mRNA levels and a tendency for a lower maximal BAT temperature

**Figure 3:**
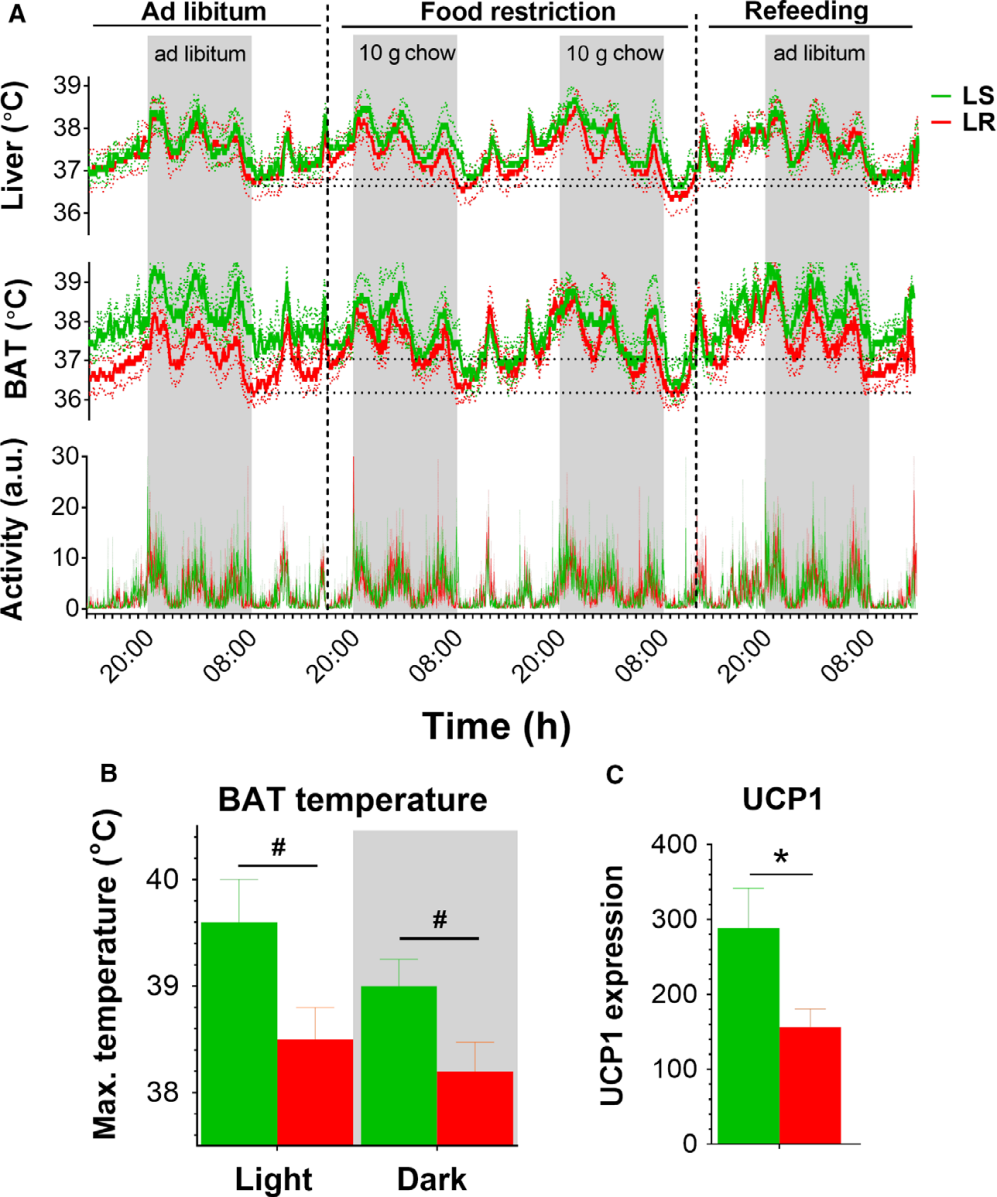
Comparison of core body and BAT temperature between LS and LR rats. (A) Core body (liver) temperature, BAT temperature, and locomotor activity during ad libitum feeding, food restriction (10 grams of chow overnight for 2 days), and refeeding in LS versus LR rats. For statistics, see Tables S2 and S3. Data are shown as mean ± SEM. The dotted lines show the SEM. N = 3–5 for LS rats and n = 5–6 for LR rats. (B) Maximal BAT temperature in the light and dark phase in LS versus LR rats during ad libitum feeding. For each rat, the average of the five highest BAT temperatures during ad libitum feeding was taken during the light phase and dark phase, respectively. Flight = 2.377, P = 0.084; Fdark = 2.203, P = 0.091. Data are shown as mean ± SEM; n = 3 for LS rats and n = 5 for LR rats. #P = 0.08‐0.09 in LS versus LR rats. The shaded areas indicate the dark phase. (C) BAT UCP1 mRNA expression (2^‐(∆ cycle threshold) with Hmbs as reference gene) in LS versus LR rats. Data are shown as mean ± SEM; n = 4 per group; t = 2.260, *P = 0.033.

We implanted intraabdominal transmitters with probes to the liver (core body temperature) and BAT (Fig. 1A), to compare thermogenesis under distinct feeding conditions (i.e., ad libitum feeding, food restriction, and refeeding) (Fig. 3A). During ad libitum feeding, LR rats showed a trend for a reduction in BAT temperature compared with LS rats (Fig. 3A, Table S2). Both during the light and dark phase, the absolute difference in BAT temperature between LS and LR rats was large, as BAT temperature of LR rats was on average 1.0 ± 0.2°C lower (dark: LS 38.5 ± 0.61°C vs. LR 37.3 ± 0.39°C; light: LS 37.8 ± 0.43°C vs. LR 36.8 ± 0.39°C). We also challenged rats by food restricting (FR) them for 2 days by giving them 10 grams of chow overnight (i.e., half of the normal amount of food intake) (Fig. 3A). LS rats gradually reduced their BAT temperature during FR, reaching their lowest body temperature at 9.00 h (1 h into the light phase) after 2 days of FR. At this time point, BAT temperature was reduced from 37.4 ± 0.47°C during ad libitum feeding to 36.4 ± 0.41°C at FR day 2 (t = 5.182, P = 0.035). In contrast to LS rats, LR rats did not reduce their BAT temperature during food restriction, as BAT temperature at 9.00 h was 36.4 ± 0.39°C during ad libitum feeding and 36.2 ± 0.36°C at FR day 2 (t = 2.998, P = 0.096). These findings suggest that the minimal BAT temperature in both LS and LR rats was around 36.2–36.4°C. Perhaps LR rats did not further reduce their BAT temperature during FR because their BAT temperature was already low during ad libitum feeding, which suggests that BAT of LR rats shows a lower thermogenic capacity. Indeed, analysis of the maximal BAT temperature in LS versus LR rats revealed that LR rats show a trend for a lower maximal BAT temperature during both the light phase (LS 39.6 ± 0.39°C vs. LR 38.5 ± 0.31°C) and the dark phase (LS 39.0 ± 0.25°C vs. LR 38.2 ± 0.28°C) when ad libitum fed (Fig. 3B). In accordance, LR rats showed significantly lower mRNA expression levels of uncoupling protein 1 (UCP1) in BAT tissue compared with LS rats (Fig. 3C), and UCP1 expression levels strongly correlated with maximal BAT temperature in both the light phase and the dark phase (R2 ≥ 0.994, P ≤ 0.049). Refeeding increased BAT temperature in both LS and LR rats, and the difference in BAT temperature between LS and LR rats gradually reinstated over time from 0.5°C during light phase 1, to 0.7°C during dark phase 1, and 0.8°C during light phase 2 (Fig. 3A, Table S2).

The large absolute differences in BAT temperature between LS and LR rats when ad libitum fed did not result in significant differences in core body (liver) temperature between LS and LR rats (Fig. 3A, Table S2). However, LR rats showed a nonsignificant reduction of 0.3 ± 0.2°C in core body temperature compared with LS rats during FR (average temperature LS 37.6 ± 0.25°C vs. LR 37.3 ± 0.45°C). LS and LR rats both significantly reduced their core body temperature after 2 days of FR (9.00 h during ad libitum feeding vs. FR day 2, LS: 36.9 ± 0.32°C vs. 36.7 ± 0.21°C, t = 4.265, P = 0.05; and LR: 36.9 ± 0.41°C vs. 36.3 ± 0.46°C, t = 7.527, P = 0.002). So, both LS and LR rats reduced their core body temperature during restricted food availability, probably to conserve energy. However, the mechanism by which they reduce their body temperature differs, as LS but not LR rats strongly reduce their BAT thermogenesis during FR. Locomotor activity followed a similar circadian pattern as BAT and core body temperature, but did not differ between the LS and LR groups (Fig. 3A, Table S3), suggesting that the temperature differences between these groups were independent of locomotor activity. Tail temperature following 2 days of FR was not different between LS and LR rats (LS 30.0 ± 0.4°C vs. LR 30.3 ± 0.3°C), as was tested during the two experiments described below.

### LR rats are resistant to the thermogenic effects of intravenous leptin

Leptin is known to robustly increase body temperature in states of low leptin levels, like fasting (Heeren and Munzberg 2013; Morrison et al. 2014; Rezai‐Zadeh et al. 2014). In order to compare leptin regulation of thermogenesis between LS and LR rats, rats were food restricted for two consecutive days, and leptin was injected around 9.00 h at FR day 2, as this was the time point at which body temperature was lowest in both LS and LR rats (Fig. 1B, 3A). Leptin‐induced thermogenesis was tested both in the absence of food and after the food was returned (refeeding). We also tested leptin‐induced heat loss via the tail by measuring tail base temperature with an infrared/thermal camera (Fig. 1A).

**Figure 4:**
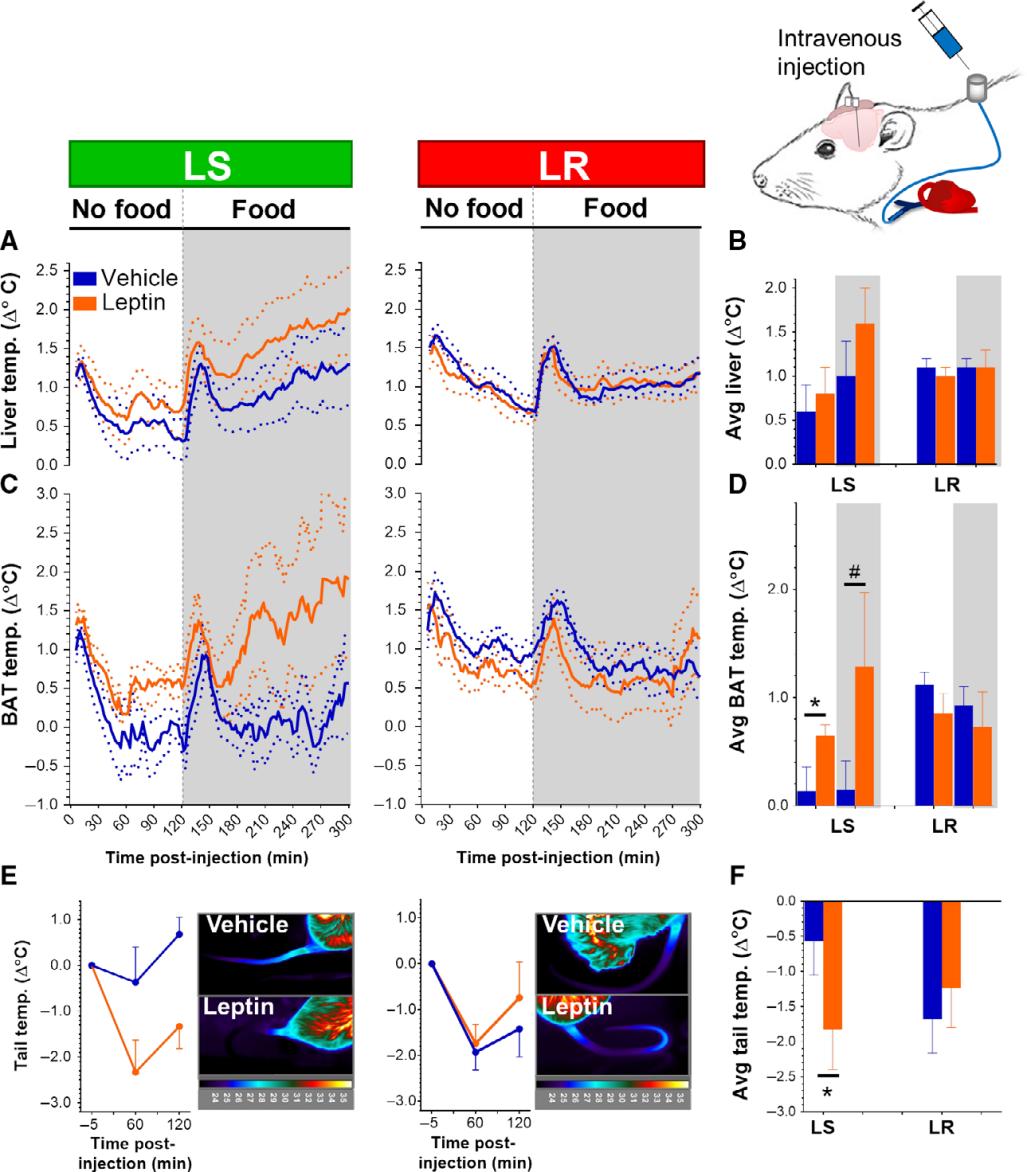
Comparison of leptin regulation of thermogenesis between LS and LR rats after intravenous leptin administration. (A) Continuous and (B) average change in core body (liver) temperature after intravenous leptin/vehicle injection in LS (n = 5) versus LR rats (n = 9), in the presence and absence of food. Without food: Ftreatment*responder=1.441, P = 0.107; with food: Ftreatment*responder=2.945, P = 0.112. (C) Continuous and (D) average change in BAT temperature after intravenous leptin/vehicle injection in LS (n = 3) versus LR rats (n = 6), in the presence and absence of food. Without food: Ftreatment*responder=4.218, P = 0.079. Post hoc in LS and LR separately: LS t = −3.474, P = 0.037; LR t = 1.052, P = 0.171. With food: Ftreatment*responder=7.961, P = 0.026. Post hoc in LS and LR separately: LS t = −2.632, P = 0.06; LR t = 0.760, P = 0.240. (E) Change in tail base temperature and representative temperature images after intravenous leptin/vehicle injection in LS (n = 9) versus LR (n = 11) rats in the absence of food. Thermal images were taken shortly before injection, and at 60 and 120 min. postinjection. (F) Average change in tail base temperature. Ftreatment*responder=3.718, P = 0.074. Post hoc in LS and LR separately: LS t = 2.086, P = 0.041; LR t = −0.791, P = 0.226. Data are shown as mean ± SEM. The dotted lines show the SEM. *P < 0.05, #P = 0.06 for leptin versus vehicle. The shaded areas indicate measurements in the presence of food.

In the absence of food, LS rats showed a tendency for a different BAT and tail base temperature response to intravenous leptin compared with LR rats (Fig. 4C–F). LS rats showed a significant leptin‐induced increase in BAT temperature (Fig. 4C and D) and a significant reduction in tail base temperature (Fig. 4E and F) compared to vehicle, indicating that thermoregulation in LS rats is leptin sensitive. The activation of these leptin‐induced thermoregulatory mechanisms in LS rats caused a slight rise in core body temperature (+0.2°C) (Fig. 4A and B), that did not reach statistical significance. After returning the food, LS rats showed a significantly different BAT temperature response to leptin compared with LR rats (Fig. 4D). Only LS rats showed a trend for a leptin‐induced increase in BAT temperature, that was greater in magnitude than in the absence of food (average change in BAT temperature with leptin compared to vehicle after food return +1.2°C vs. +0.6°C before food return), and was accompanied by a nonsignificant increase in core body temperature of +0.6°C. In contrast to LS rats, LR rats did not show any effect of leptin on BAT, tail base, and core body temperature (Fig. 4A–F), which indicates that LR rats are not only resistant to leptin's anorexigenic effects, but also to leptin's thermoregulatory effects. The differences in body temperature regulation by leptin in LS versus LR rats were independent of locomotor activity (Fig. S3).

### LR rats show defective temperature regulation in the DMH

**Figure 5:**
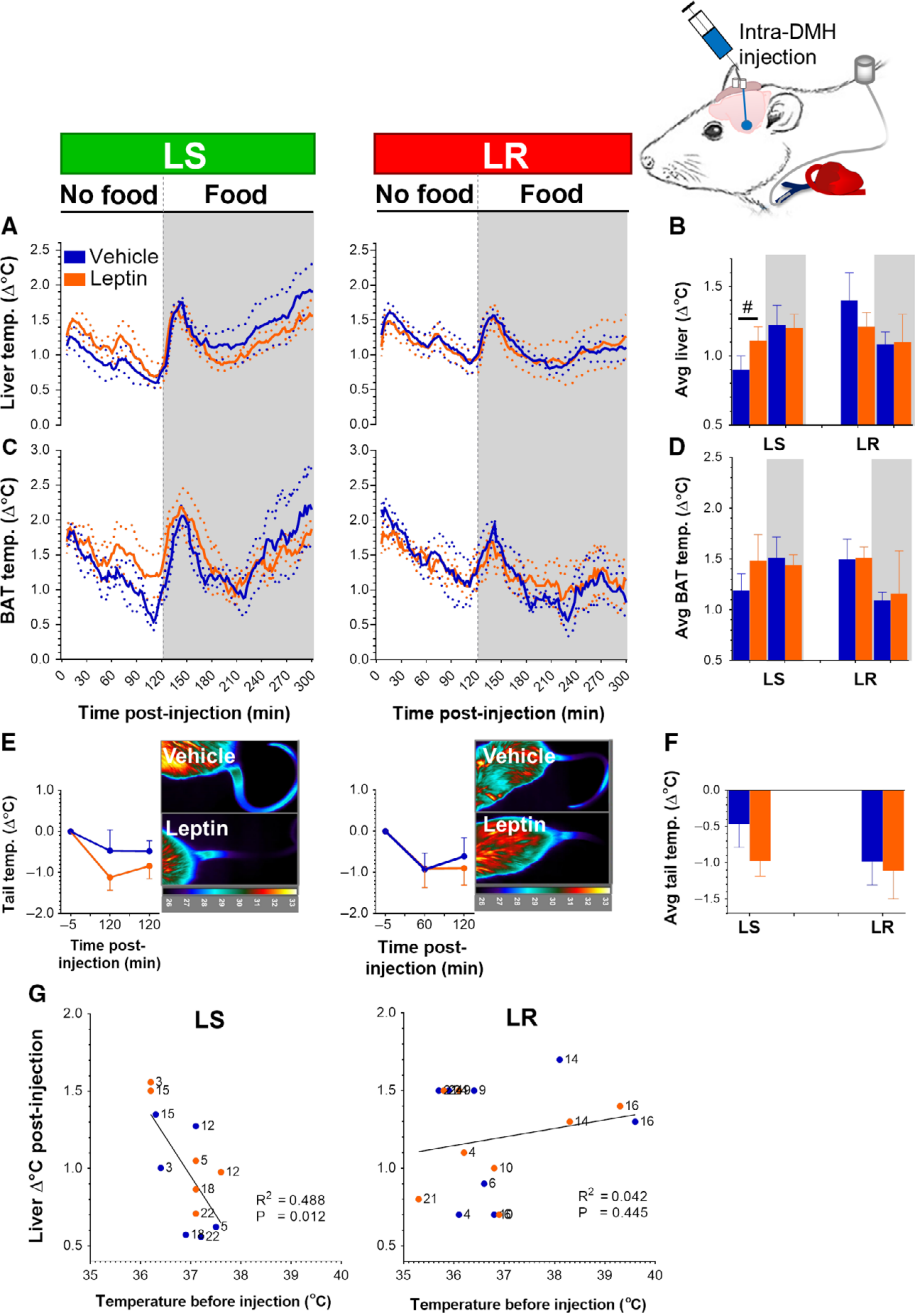
Comparison of leptin regulation of thermogenesis between LS and LR rats after intra‐DMH leptin administration. (A) Continuous and (B) average change in core body (liver) temperature after intra‐DMH leptin/vehicle injection in LS (n = 6) versus LR rats (n = 7), in the presence and absence of food. Without food: Ftreatment*responder=1.973, P = 0.186; with food: Ftreatment*responder=0.389, P = 0.544. (C) Continuous and (D) average change in BAT temperature after intra‐DMH leptin/vehicle injection in LS (n = 3) versus LR rats (n = 4), in the presence and absence of food. Without food: Ftreatment*responder=2.214, P = 0.187; with food: Ftreatment*responder=0.009, P = 0.928. (E) Change in tail base temperature and representative temperature images after intra‐DMH leptin/vehicle injection in LS (n = 7) versus LR (n = 9) rats in the absence of food. Thermal images were taken shortly before injection, and at 60 and 120 min. postinjection. (F) Average change in tail base temperature. Ftreatment*responder=0.273, P = 0.608. (G) Correlation between core body temperature before injection and delta change in core body temperature 0–2 h following injection of leptin (orange) or vehicle (blue). LS, R2 = 0.488, P = 0.012, and LR, R2 = 0.042, P = 0.445. Data are shown as mean ± SEM. The dotted lines show the SEM. #P = 0.07 for leptin versus vehicle. The shaded areas indicate measurements in the presence of food.

We used the same approach to that used for intravenous leptin to compare the effects of intra‐DMH leptin injection on body temperature in the two groups. LS and LR rats did not significantly differ in the effect of leptin on core body temperature (Fig. 5A and B), BAT temperature (Fig. 5C and D), tail base temperature (Fig. 5E and F), and locomotor activity (Fig. S4), in either the absence or presence of food. Closer inspection of the thermoregulatory effects of leptin in LS rats in the absence of food, revealed that the direction of the effect of leptin on BAT and tail base temperature was similar compared with that after intravenous injection, but the effect sizes were smaller (intra‐DMH vs. intravenous leptin injection, BAT: 0.3°C vs. 0.6°C; tail base: −1.2°C vs. −0.5°C). The smaller effect of intra‐DMH leptin on thermoregulatory mechanisms resulted in a similar trend for an increase in core body temperature as with intravenous leptin (both +0.2°C), which suggests that the thermoregulatory responses evoked by enhanced leptin signaling in the DMH are more effective in modulating core body temperature than those evoked by systemic induction of leptin signaling. LR rats did not show any increase in BAT and core body temperature or a reduction in tail base temperature with intra‐DMH leptin (Fig. 5A–F), suggesting that leptin resistance in the DMH could contribute to the failure of leptin to affect thermoregulation in LR rats.

We noticed that core body temperature before injection was very variable between (but not within) individual rats, as it ranged from 36.1 to 37.2°C in LS rats to even 35.3–39.6°C in LR rats before intra‐DMH injection (Fig. 5G). Since leptin has been suggested to have a permissive rather than active thermogenic effect (Heeren and Munzberg 2013; Rezai‐Zadeh and Munzberg 2013; Morrison et al. 2014), we compared baseline core body temperature with delta temperature change after leptin and vehicle injection. In LS rats, we found a significantly negative correlation between body temperature before injection, and delta temperature change following intra‐DMH injection (Fig. 5G), indicating a reduced temperature response when core body temperature was relatively high before injection. All individual LS rats, except for one, showed a larger delta temperature change with leptin compared with vehicle. In contrast, LR rats did not show a correlation between core body temperature before injection and delta temperature change after leptin injection, illustrating a defect in the coupling between core body temperature and the facilitation of thermoregulatory mechanisms by leptin.

## Discussion

As in humans, rats show high variability in the susceptibility to develop obesity (Levin and Dunn‐Meynell 2002; Levin et al. 2004; Tulipano et al. 2004; de Git et al. 2018). Based on the feeding response to leptin on a chow diet, rats were divided into leptin‐sensitive (LS) and leptin‐resistant (LR) groups. We here show that LS rats show a thermogenic response to leptin, which appears to be exerted, at least in part, at the level of the DMH. In contrast, LR rats did not show any thermogenic response to leptin, thereby linking their preexisting reduction in pSTAT3 activation in the DMH (de Git et al. 2018) to impaired leptin regulation of thermogenesis.

Leptin is thought to influence BAT thermogenesis in a permissive manner rather than being actively thermogenic, that is, it probably signals the availability of lipid and glucose fuel supplies for oxidation in BAT, and acts through LepRb to enhance the excitability of neurons controlling BAT activity, thereby facilitating BAT activation (Heeren and Munzberg 2013; Rezai‐Zadeh and Munzberg 2013; Morrison et al. 2014). Therefore, resistance to leptin likely contributes to the 1°C lower basal BAT temperature, the lower maximal BAT temperature, and the lower BAT UCP1 levels in LR rats compared with LS rats under ad libitum feeding. During food restriction, circulating leptin levels are low, which may result in lower BAT activity (Heeren and Munzberg 2013; Morrison et al. 2014; Rezai‐Zadeh et al. 2014). A reduction in BAT thermogenesis was indeed observed in LS but not LR rats during food restriction, which was restored during refeeding. Perhaps BAT temperature was already at its lowest point during ad libitum feeding in LR rats due to leptin resistance. Since BAT thermogenesis has the capacity to prevent excess body weight gain in response to an obesogenic diet (Rezai‐Zadeh and Munzberg 2013), the preexisting reduction in BAT thermogenesis in LR rats likely predispose them to exacerbated obesity on a fcHFHS diet.

Besides compelling evidence for a BAT‐dependent thermogenic increase in core body temperature with leptin (Himms‐Hagen 1985; Commins et al. 1999, 2001; Enriori et al. 2011; Rezai‐Zadeh et al. 2014), leptin was recently shown to lead to a pyrexic increase in core body temperature by reducing heat loss via the tail in ob/ob mice, without affecting BAT thermogenesis (Fischer et al. 2016b). After intravenous leptin injection, LS rats significantly increased their BAT thermogenesis and reduced their heat loss via the tail, resulting in a modest increase in core body temperature. This phenomenon has been observed previously for BAT thermogenesis (Enriori et al. 2011). BAT temperature was proposed to be a more sensitive marker for the sympathoexcitatory effects of leptin than total body temperature (Enriori et al. 2011). Rather than one fixed body temperature set point, different thermoregulatory mechanisms (such as BAT thermogenesis and tail vein constriction) may have different thresholds that need not be synchronized (Fischer et al. 2016a).

Using a variety of approaches, leptin action in the DMH has previously been shown to play a critical role in BAT‐dependent thermogenesis (Enriori et al. 2011; Dodd et al. 2014; Rezai‐Zadeh et al. 2014). In the absence of food, we found a small, nonsignificant increase in BAT temperature with intra‐DMH leptin in LS rats. Enriori et al. (2011) also tested the effect of intra‐DMH leptin injection on BAT temperature, and found a potent induction of BAT thermogenesis. We speculate that differences in injection volume may explain the discrepancy with the above described results of Enriori et al., as they injected 0.5 μL (0.2 μg/μL) leptin into the DMH of mice, whereas we injected 0.3 μL (1 μg/μL) leptin into the DMH of rats. Leakage of leptin into surrounding hypothalamic nuclei, which were also shown to mediate the effect of leptin on BAT activation (Enriori et al. 2011; Morrison et al. 2014; Pandit et al. 2017), could explain the stronger effect of leptin on BAT thermogenesis in mice in the study of Enriori et al. Even though the effects of intra‐DMH leptin on BAT and tail temperature were small in LS rats, they were in the same direction as with intravenous leptin, and led to a similar trend for an increase (+0.2°C) in core body temperature. This suggests that the thermoregulatory mechanisms activated by leptin signaling in the DMH are more directly aimed at increasing core body temperature. Alternatively, as leptin may permissively increase core body temperature until the body temperature set point is achieved (Heeren and Munzberg 2013; Rezai‐Zadeh and Munzberg 2013; Morrison et al. 2014), the thermoregulatory mechanisms evoked by intra‐DMH leptin may have been sufficient to raise core body temperature to this set point. As such, the larger thermoregulatory changes in BAT and tail temperature with intravenous leptin did not further raise core body temperature compared to that with intra‐DMH leptin, as small thermoregulatory changes were sufficient to achieve the body temperature set‐point.

One limitation of this study is that we focused on effects of leptin on different measures of thermogenesis which took into account that at least some of these effects were mediated via the DMH. Other routes via which leptin affects temperature, such as via the preoptic area or via thyroid axis (de Vries et al. 2015; Deem et al. 2018) were not investigated but could contribute to mediate the different sensitivity for leptin in LS versus LR rats. Another limitation of this study is the difference in group size and the low number of animals in the LS group. The LR rats, being the larger group, did not show any thermogenic response to leptin. Since we found the distinction in leptin sensitivity between LS and LR rats both at the level of food intake and thermogenesis, we believe that the thermogenic effects we observed in LS rats are valid. For intra‐DMH injections, we showed that LS rats were less capable of increasing their core body temperature with leptin when their body temperature was relatively high before injection. Although this correlation nicely demonstrates that leptin permissively increases core body temperature in LS rats until the set point for core body temperature is achieved, it also explains the large variability in the thermogenic response to leptin between rats. The finding that LR rats did not show a coupling between baseline core body temperature and the temperature response to intra‐DMH leptin, further supports impaired thermoregulation by leptin signaling in the DMH in LR rats. However, we cannot exclude that some differences in leptin‐induced thermoregulation between LS and LR rats reflect variability in injection stress sensitivity. Higher stress‐induced hyperthermia following injection of vehicle and leptin in LR rats might explain why LR rats do not respond to leptin.

To conclude, our study shows that leptin increases BAT thermogenesis and reduces heat loss via the tail in LS rats. LR rats show a preexisting resistance to these thermoregulatory effects of leptin, which seems to be mediated via leptin resistance exerted, at least in part, at the level of the DMH. This resistance may explain the lower BAT capacity under ad libitum feeding which may, in turn, predispose LR rats to exacerbated obesity when exposed to an obesogenic fcHFHS diet. Future studies are necessary to determine whether LR rats are indeed not able to sufficiently adapt their BAT thermogenesis to the increased caloric intake on a fcHFHS diet, and whether this is the mechanism by which they become excessively obese. Altogether, these data illustrate that reduced leptin regulation of thermogenesis may be a mechanism that explains how a preexisting reduction in leptin sensitivity in the DMH predisposes rats to exacerbated obesity.

## Conflict of interest

The authors declare that they have no conflict of interest.

## Supporting information




**Figure S1.** Obesity‐related parameters in LS and LR rats on a chow diet.
**Figure S2.** Individual 1–24h leptin sensitivity following intra‐DMH leptin injection in LS and LR rats.
**Figure S3.** Comparison of leptin regulation of locomotor activity between LS and LR rats after intravenous leptin administration.
**Figure S4.** Comparison of leptin regulation of locomotor activity between LS and LR rats after intra‐DMH leptin administration.
**Table S1.** Overview of the re‐use of rats in BAT, liver, and tail temperature measurements.
**Table S2.** Statistical analysis of BAT and liver temperature in LS versus LR rats.
**Table S3.** Statistical analysis of activity in LS versus LR rats.
