# Supplementary material for: Rats that are predisposed to excessive obesity show reduced (leptin‐induced) thermoregulation even in the preobese state
Source: Physiol Rep. 2019 Jul 24;7(14):e14102. doi: 10.14814/phy2.14102 (PMC6656864; doi:10.14814/phy2.14102)
Supplement: Supplementary file 1 — Figure S1. Obesity‐related parameters in LS and LR rats on a chow diet. Figure S2. Individual 1–24h leptin sensitivity following intra‐DMH leptin injection in LS and LR rats. Figure S3. Comparison of leptin regulation of locomotor activity between LS and LR rats after intravenous leptin administration. Figure S4. Comparison of leptin regulation of locomotor activity between LS and LR rats after intra‐DMH leptin administration. Table S1. Overview of the re‐use of rats in BAT, liver, and tail temperature measurements. Table S2. Statistical analysis of BAT and liver temperature in LS versus LR rats. Table S3. Statistical analysis of activity in LS versus LR rats. [file PHY2-7-e14102-s001.docx]

***Supplementary information***

**Rats that are predisposed to excessive obesity show reduced leptin-induced thermoregulation before becoming obese**

Kathy C.G. de Git^1^, Johannes A. den Outer^1^, Inge G. Wolterink-Donselaar^1^, Mieneke C.M. Luijendijk^1^, Erik Schéle^2^, Suzanne L. Dickson^2^, Roger A.H. Adan^1,2*^

1) Brain Center Rudolf Magnus, Dept. of Translational Neuroscience, University Medical Center Utrecht, Utrecht University, Utrecht 3584 CG, The Netherlands

2) Institute for Neuroscience and Physiology, The Sahlgrenska Academy at the University of Gothenburg, SE-405 30 Gothenburg, Sweden

**^*^Corresponding author**

Roger A.H. Adan, PhD

Professor of Molecular Pharmacology

Department of Translational Neuroscience, Brain Center Rudolf Magnus

University Medical Center Utrecht, Utrecht, 3584 CG, the Netherlands

Email: r.a.h.adan@umcutrecht.nl

Tel: (+31) 88 756 8517

**Methods**

**Surgery**

Surgery was performed under fentanyl/fluanisone (0.315 mg/kg fentanyl, 10 mg/kg fluanisone, Hypnorm, Janssen Pharmaceutica, Beerse, Belgium) and midazolam (2.5 mg/kg, i.p., Actavis, the Netherlands) anesthesia. Xylocaine was sprayed on the skull to provide local anesthesia (Lidocaine 100 mg/ml, AstraZeneca BV, Zoetermeer, the Netherlands). All rats received three daily peri-surgical injections of carprofen (5 mg/kg, s.c. Carporal, AST Farma BV, Oudewater, the Netherlands), starting at the day of surgery.

**Selection of leptin sensitive vs resistant rats**

To measure leptin sensitivity, animals were fasted overnight (10 gr chow at 16.00h). The next morning at 10.00h, leptin (250 µg / 250 µl; recombinant murine leptin, NHPP, USA) or vehicle (250 µl, phosphate buffered saline, PBS) was injected via a jugular vein catheter, and 45 minutes later food was made available once again. A latin-square design was used, such that half of the rats first received leptin, and 3-4 days later, were tested a second time with treatments reversed. Food intake was measured 1-24h after food return using an automated food-monitoring system (Scales, Department Biomedical Engineering, UMC Utrecht, The Netherlands). This program records the weight of food hoppers in the home cage automatically every 12 s. Leptin sensitivity was measured by normalizing cumulative food intake after leptin injection to cumulative food intake after vehicle injection. In each individual rat, two independent leptin sensitivity tests were performed, and subsequently the average response of two tests was taken (Fig. 2A).

In six rats, the response to leptin in test 1 largely deviated from the response in test 2, which could be explained by external factors (reasons for deviation included the bleeding of cannulas following injection, stress effects, and abnormal water/food intake during the days before injection). These rats were tested a third time, and the response of the third test was averaged with the response of the most reliable test from the first two tests, which made it possible to reliably designate rats as LS or LR.

**Thermosensitive camera**

In order to make thermal photographs, rats were placed in an open cage to which they were extensively habituated. The camera was mounted approximately 50 cm above the cage, and thermal photographs focused on the tail base. A thermal photograph was made shortly before injection (baseline), and at one and two hours following injection. For each time-point, several photographs were taken and the photograph with best tail base visibility was analyzed. Photographs were analyzed with a specific software package (FLIR-Tools-Software; FLIR; West Malling, Kent, UK). The region of interest covered the start of the tail base (Fig. 1A), and the center temperature measurement was used for the analysis.

**Post-mortem analysis**

In order to check for cannula placement, rats were given a lethal dose of sodium pentobarbital (200 mg/ml, Euthanimal, Alfasan BV, Woerden, The Netherlands), and were transcardially perfused with 0.9% NaCl followed by 4% paraformaldehyde (PFA) in PBS. Brains were excised and kept in 4% PFA for 24h, and were subsequently saturated with 30% sucrose in PBS with 0.01% NaN_3_. Brains were snap frozen in isopentane between -60ºC and -40ºC, and sliced into 40 µm sections using a cryostate (Leica, Germany). Tissue was collected in six series in cryo-protectant (25% glycerol; 25% ethylene-glycol in PBS) and stored at -20ºC. Two series were mounted, and subsequently photographed and digitized using a Zeiss Axioskop 2 microscope (Zeiss, Jena, Germany). Slices were matched to the stereotaxic brain atlas from Paxinos and Watson (1998; fourth edition), using the fornix, mammillothalamic tract, and optic tract as landmarks for the DMH, in order to check for guide cannula placement above the DMH (Fig. 1C).

**Body composition**

Prior to perfusion, individual epididymal, subcutaneous (inguinal), mesenteric, and perirenal white adipose tissues were dissected from the left side, cleaned and weighed.

**Uncoupling protein 1 expression**

Two hours before sacrifice, all chow was removed and rats were bilaterally injected with either 300 ηl PBS (4 LS and 4 LR rats) or 300 ηg leptin / 300ηl PBS leptin (remaining rats) in the DMH. Shortly before perfusion, BAT tissue was dissected and stored at -80ºC. Only BAT tissue of PBS injected rats was used to determine uncoupling protein 1 (UCP1) expression levels.

*RNA extraction and cDNA synthesis*

Frozen BAT samples were homogenized in QIAzol lysis reagent using a Tissue Lyser (Qiagen, Hilden, Germany) and chloroform was subsequently added to the homogenates prior to centrifugation at 4°C. RNA was isolated from the received upper aqueous phase using RNeasy Lipid Tissue Mini kit (Qiagen). DNA was removed by DNase treatment (Qiagen). Acquired RNA was diluted to a concentration of 50 ng/µl and reversed transcribed by using iScript cDNA synthesis kit (Bio-Rad laboratories, Hercules, CA, USA).

*Quantitative real-time polymerase chain reaction (qRT-PCR) analysis*

PCR reaction mixture was prepared by mixing 25 ng of cDNA with TaqMan Advanced Master Mix (Applied Biosystems, Carlsbad, CA, USA) and TaqMan Gene Expression Assay (Ucp1; Rn00562126_m1, Hmbs; Rn00565886_m1). Thermal cycling and fluorescence detection was performed with a QuantStudio 7 Flex Real-Time PCR System (Applied Biosystems). The cycle threshold (Ct) was set automatically by the system software. The Ucp1 Ct values were normalized to the reference gene Hmbs. Relative mRNA expression levels were calculated by using the 2-∆Ct equation, where the ∆Ct value was obtained by subtracting Ct value of the reference gene Hmbs from the Ct value of Ucp1.

**Figures**

**Fig.S1**

**Fig. S1** Obesity-related parameters in LS and LR rats on a chow diet. (A) Body weight and (B) caloric intake. F_week*responder_≥0.610, p≥0.392. (C) EWAT, epididymal; SWAT, subcutaneous (inguinal); PWAT, perirenal, and MWAT, mesenteric white adipose tissues at week 15. F_responder_≥0.099, p≥0.168. Data are shown as mean ± SEM. N=10-11 per group.

**Fig. S2**

**
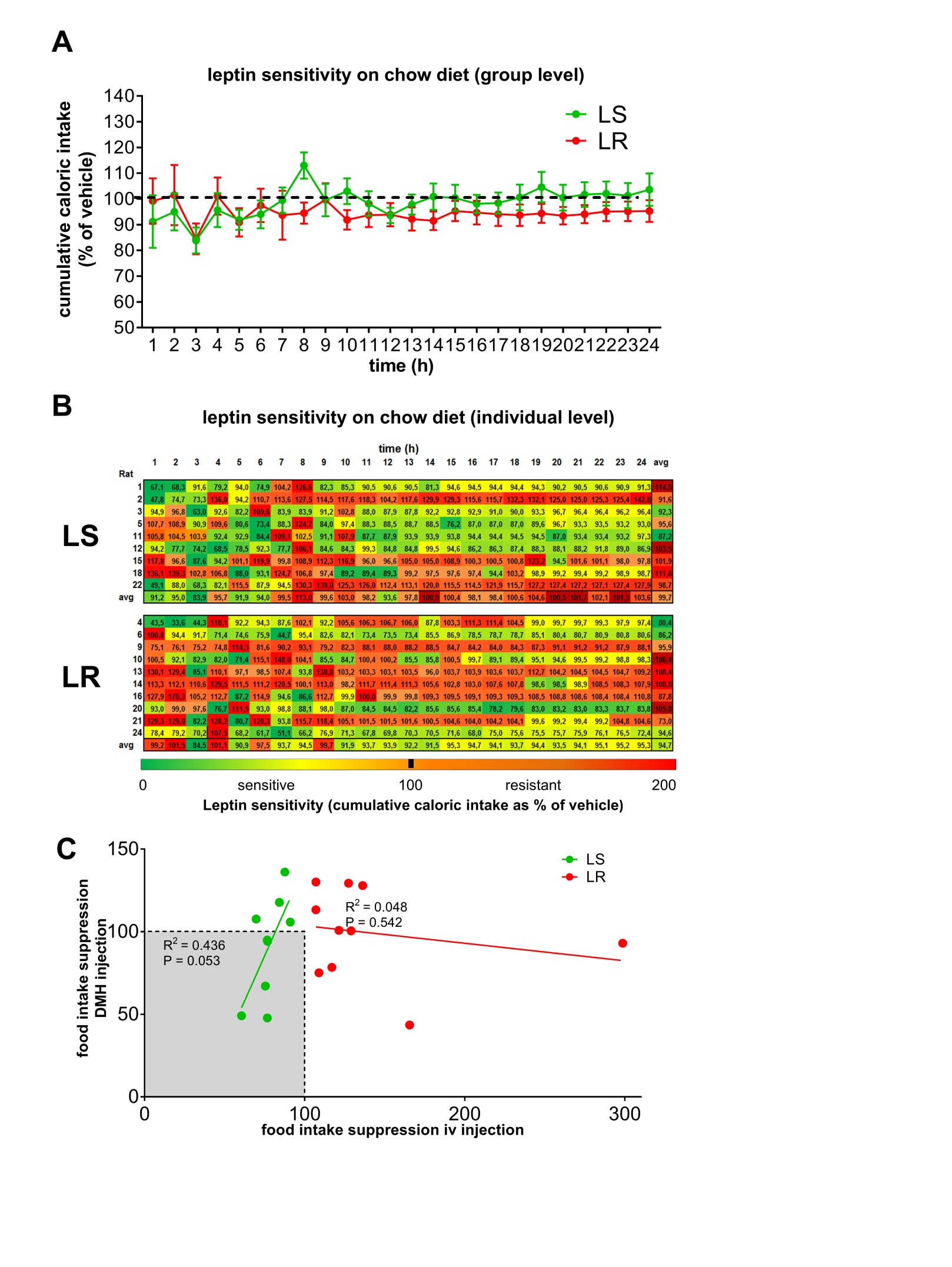
**

**Fig. S2** Individual 1-24h leptin sensitivity following intra-DMH leptin injection in LS and LR rats. Leptin sensitivity was measured by cumulative food intake after leptin injection normalized to vehicle food intake. Leptin sensitivity of one test is shown. Leptin sensitivity (A) at group level and (B) individual level; a heat plot of the relative level of sensitivity is shown at 1-24h food intake for each individual rat (i.e. each row). The heat plot indicates the relative degree of leptin sensitivity at a particular time point in comparison with the other time-points in the row. 1-24h: F_(hour*)treatment*responder_≥0.597, p≥0.422. LS, F_treatment*hour_=1.595, p=0.049; F_treatment_=0.161, p=0.699. LR, F_treatment*hour_=0.754, p=0.785; F_treatment_=1.792. p=0.214. Data are shown as mean ± SEM; n=9-10 per group (C) Correlation between food intake suppression 1h following intravenous and intra-DMH leptin injection. LS, R^2^=0.436, p=0.053, and LR, R^2^=0.048, p=0.542.

**Fig. S3**

**Fig. S3** Comparison of leptin regulation of locomotor activity between LS and LR rats after intravenous leptin administration. (A) Continuous and (B) average delta change in locomotor activity after intravenous leptin/vehicle injection in LS (n=5) vs LR rats (n=9), in the presence and absence of food. Without food: F_treatment*responder_=0.625, p=0.445; with food: F_treatment*responder_=1.047, p=0.326. Data are shown as mean ± SEM. The dotted lines show the SEM. The shaded areas indicate measurements in the presence of food.

**Fig. S4**

**Fig. S4** Comparison of leptin regulation of locomotor activity between LS and LR rats after intra-DMH leptin administration. (A) Continuous and (B) average delta change in locomotor activity after intra-DMH leptin/vehicle injection in LS (n=6) vs LR rats (n=9), in the presence and absence of food. Without food: F_treatment*responder_=0.149, p=0.706; with food: F_treatment*responder_=1.891, p=0.194. Data are shown as mean ± SEM. The dotted lines show the SEM. The shaded areas indicate measurements in the presence of food.

**Tables**

**Table S1. Overview of the re-use of rats in BAT, liver, and tail temperature measurements.**


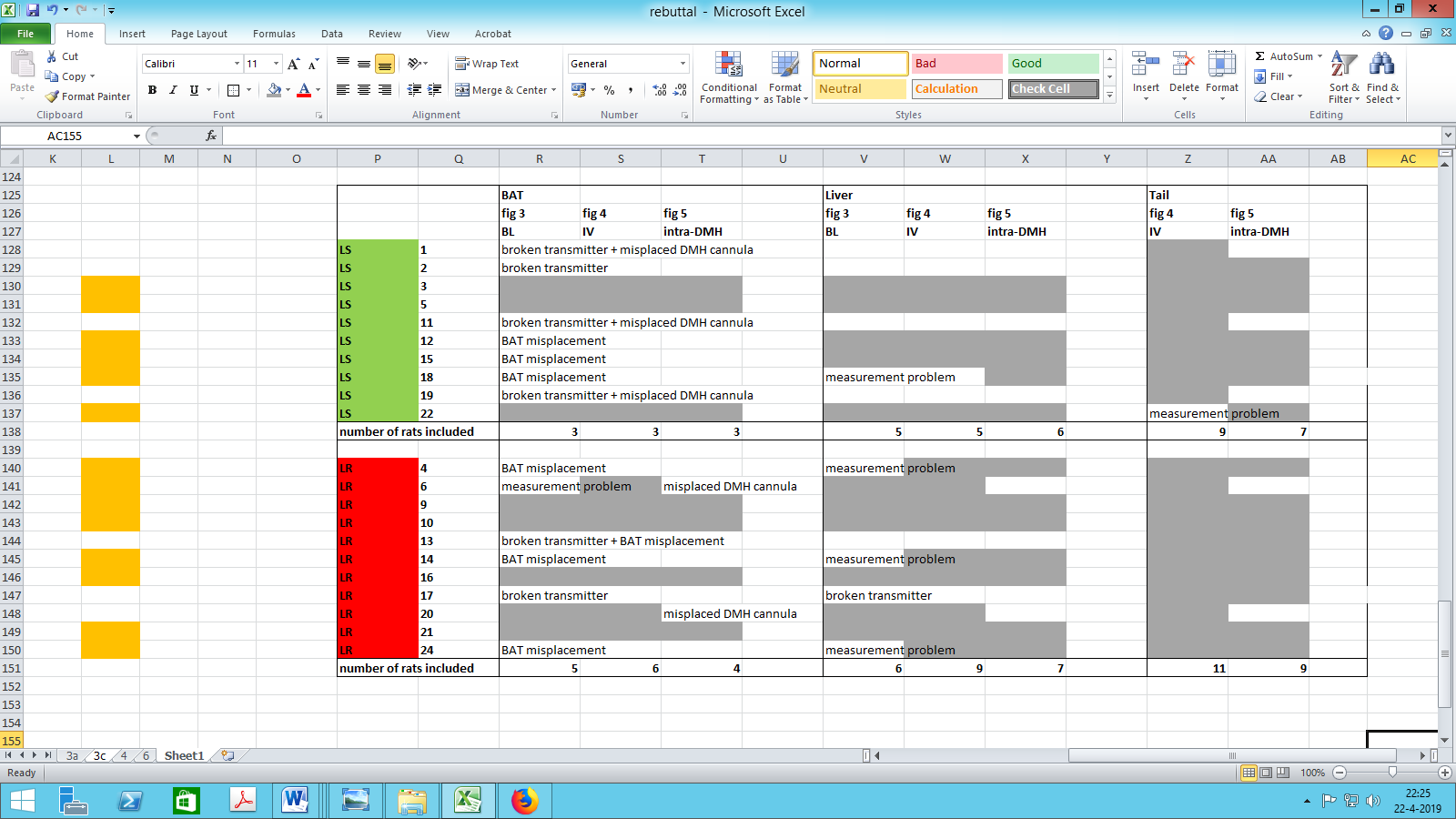


Rats were grouped into leptin sensitive (LS) and leptin resistant (LR) rats. Grey shading indicates tests in which rats were included. The reasons for exclusion from one or multiple tests are indicated for each individual rat.

**Table S2.** Statistical analysis of BAT and liver temperature in LS versus LR rats.

|  |  | **LS vs LR test**  **Statistic** | |  | **P value**  Two-sided One-sided | |
| --- | --- | --- | --- | --- | --- | --- |
| **BAT temperature** \ | | | | | | |
| **Ad libitum feeding** | | | | | | |
| Light (total) | | | F_time*responder_ = 1.619  F_responder_ = 2.463 |  | **0.087^#^**  0.161 | **0.081^#^** |
| Dark | | | F_time*responder_ = 0.620  F_responder_ = 2.528 |  | 0.806  0.156 | **0.078^#^** |
| **Restricted feeding** | | | | | | |
| Light 1 | | | F_time*responder_ = 1.198  F_responder_ = 0.761 |  | 0.325  0.406 | 0.206 |
| Dark 1 | | | F_time*responder_ = 0.417  F_responder_ = 0.690 |  | 0.945  0.430 | 0.215 |
| Light 2 | | | F_time*responder_ = 0.896  F_responder_ = 0.214 |  | 0.337  0.661 | 0.331 |
| Dark 2 | | | F_time*responder_ = 0.281  F_responder_ = 0.109 |  | 0.592  0.750 | 0.375 |
| Light 3 | | | F_time*responder_ = 1.416  F_responder_ = 0.513 |  | 0.271  0.494 | 0.247 |
| **Refeeding** | | | | | | |
| Light 1 | | | F_time*responder_ = 1.477  F_responder_ = 0.457 |  | 0.202  0.524 | 0.262 |
| Dark 1 | | | F_time*responder_ = 0.587  F_responder_ = 1.059 |  | 0.833  0.343 | 0.172 |
| Light 2 | | | F_time*responder_ = 0.104  F_responder_ = 1.488 |  | 0.839  0.268 | 0.134 |
| **Liver temperature** \ | | | | | | |
| **Ad libitum feeding** | | | | | | |
| Light (total) | | | F_time*responder_ = 1.430  F_responder_ = 0.221 |  | 0.247  0.651 | 0.326 |
| Dark | | | F_time*responder_ = 0.719  F_responder_ = 0.135 |  | 0.588  0.723 | 0.362 |
| **Restricted feeding** | | | | | | |
| Light 1 | | | F_time*responder_ = 1.343  F_responder_ = 0.057 |  | 0.277  0.841 | 0.421 |
| Dark 1 | | | F_time*responder_ = 1.072  F_responder_ = 0.027 |  | 0.387  0.873 | 0.437 |
| Light 2 | | | F_time*responder_ = 1.059  F_responder_ = 0.020 |  | 0.391  0.890 | 0.445 |
| Dark 2 | | | F_time*responder_ = 1.069  F_responder_ = 0.042 |  | 0.390  0.840 | 0.420 |
| Light 3 | | | F_time*responder_ = 1.626  F_responder_ = 0.002 |  | 0.218  0.965 | 0.483 |
| **Refeeding** | | | | | | |
| Light 1 | | | F_time*responder_ = 1.360  F_responder_ = 0.248 |  | 0.233  0.628 | 0.314 |
| Dark 1 | | | F_time*responder_ = 1.365  F_responder_ = 0.239 |  | 0.225  0.633 | 0.317 |
| Light 2 | | | F_time*responder_ = 1.250  F_responder_ = 0.243 |  | 0.299  0.631 | 0.316 |
| P values in bold^#^ indicate trends for statistical differences between LS and LR rats. The one-sided test statistics for responder is also shown, as we expected lower body temperature in leptin resistant (LR) rats compared with leptin sensitive (LS) rats. | | | | | | |

**Table S3.** Statistical analysis of activity in LS vs LR rats.

|  |  | **LS vs LR test**  **statistic** | |  | **P value**  Two-sided |
| --- | --- | --- | --- | --- | --- |
| **Activity** \ | | | | | |
| **Ad libitum feeding** | | | | | |
| Light (total) | | | F_time*responder_ = 1.448  F_responder_ = 0.151 |  | 0.143  0.704 |
| Dark | | | F_time*responder_ = 0.624  F_responder_ = 0.002 |  | 0.655  0.966 |
| **Restricted feeding** | | | | | |
| Light 1 | | | F_time*responder_ = 3.674  F_responder_ = 0.059 |  | 0.019  0.812 |
| Dark 1 | | | F_time*responder_ = 0.672  F_responder_ = 0.015 |  | 0.764  0.904 |
| Light 2 | | | F_time*responder_ = 2.794  F_responder_ = 0.086 |  | 0.001  0.774 |
| Dark 2 | | | F_time*responder_ = 0.682  F_responder_ = 0.050 |  | 0.613  0.827 |
| Light 3 | | | F_time*responder_ = 1.809  F_responder_ = 0.832 |  | 0.173  0.378 |
| **Refeeding** | | | | | |
| Light 1 | | | F_time*responder_ = 1.259  F_responder_ = 0.902 |  | 0.282  0.363 |
| Dark 1 | | | F_time*responder_ = 0.730  F_responder_ = 0.048 |  | 0.511  0.831 |
| Light 2 | | | F_time*responder_ = 0.154  F_responder_ = 2.397 |  | 0.240  0.150 |

LS, leptin sensitive; LR, leptin resistant rat.
